# The development, evaluation, performance, and validation of micro-PCR and extractor for the quantification of HIV-1 &-2 RNA

**DOI:** 10.1038/s41598-024-56164-5

**Published:** 2024-04-15

**Authors:** Shyam Prakash, Ram Aasarey, Priyatma, Meenakshi Sharma, Shahid Khan, Medha

**Affiliations:** Department of Laboratory Medicine, All India Institute of Medical Sciences, Room No 11, 2nd Floor, Ansari Nagar, New Delhi, 110029 India

**Keywords:** Micro-PCR, HIV-1, HIV-2, Truenat™, TruePrep™, Biological techniques, Medical research

## Abstract

HIV infection has been a global public health threat and overall reported ~ 40 million deaths. Acquired immunodeficiency syndrome (AIDS) is attributed to the retroviruses (HIV-1/2), disseminated through various body fluids. The temporal progression of AIDS is in context to the rate of HIV-1 infection, which is twice as protracted in HIV-2 transmission. Q-PCR is the only available method that requires a well-developed lab infrastructure and trained personnel. Micro-PCR, a portable Q-PCR device, was developed by Bigtec Labs, Bangalore, India. It is simple, accurate, fast, and operationalised in remote places where diagnostic services are inaccessible in developing countries. This novel micro-PCR determines HIV-1 and HIV-2 viral load using a TruePrep™ extractor device for RNA isolation. Five ml blood samples were collected at the blood collection centre at AIIMS, New Delhi, India. Samples were screened for serology, and a comparison of HIV-1/2 RNA was done between qPCR and micro-PCR in the samples. The micro-PCR assay of HIV-RNA has compared well with those from real-time PCR (r = 0.99, i < 0.002). Micro-PCR has good inter and intra-assay reproducibility over a wide dynamic range (1.0 × 10^2^–1.0 × 10^8^ IU/ml). The linear dynamic range was 10^2^–10^8^ IU/ml. The clinical and analytical specificity of the assay was comparable, i.e., 100%. Intra-assay and inter-assay coefficients of variation ranged from 1.17% to 3.15% and from 0.02% to 0.46%, respectively. Moreover, due to the robust, simple, and empirical method, the Probit analysis has also been done for qPCR LODs to avoid uncertainties in target recoveries. The micro-PCR is reliable, accurate, and reproducible for early detection of HIV-1 and HIV-2 viral loads simultaneously. Thus, it can easily be used in the field and in remote places where quantification of both HIV-1/2 is not reachable.

## Introduction

Human immunodeficiency virus (HIV) remains a substantial public health concern, with more than 39 million people living with HIV and, as of now, no cure for HIV, including opportunistic infections globally. It targets the body's white blood cells, weakening the immune system^1^. Around 6,30,000 deaths have been reported due to HIV-related causes, and 1.3 million people have acquired HIV in 2022. HIV-1 and HIV-2 are significant challenges in countries confined to regions of West Africa, India, Western Europe, Brazil, and many other regions^2–4^. When compared to HIV-1 infections, HIV-2 infections proceed at a slower rate, affecting 20–30% of infected individuals^5^. This shows that a lesser proportion of people infected with HIV-2 experience faster disease progression than those infected with HIV-1. Being left untreated with HIV-2 can lead to a higher risk of HIV transmission, higher cost of medical management, and increased mortality.

Moreover, the frequent cross-resistance and incomplete information about the resistance pathways limit the treatment options for HIV-1 and HIV-2 infections. The discovery and global roll-out of rapid diagnostics and effective antiretroviral therapies are being considered to reduce mortality and morbidity in groups of individuals requiring lifelong viral suppressive therapy. In Asia, 95% of HIV-2 infected cases come from are reported in India^6^. The prevalence of HIV-2 in India varies regionally, ranging between 2 and 33% of all HIV infections. These reports confirm infection serologically^6,7^. The frequency of HIV-2 in blood donors was reported at 2.8% (1.3% HIV-2 and 1.5% HIV-1/2 dual infection) of all HIV diagnoses at a tertiary referral hospital in southern India^1^. RNA testing is the approved diagnostic method for HIV screening of HIV-1/HIV-2 Ab combo assay in the sample. However, the difference between HIV-1/HIV-2 infection according to CDC guidelines still needs to be resolved. Therefore, for HIV prevention, treatment, and early care of HIV infection, urgently needs to assess the HIV-1 and HIV-2 detection in blood samples. Quantitative nucleic acid detection assays are primarily based on real-time PCR and are applicable for detecting molecular pathogens in diagnostics. They have developed as a crucial and valuable application, resulting in substantial advances in molecular diagnostics. Quantitative nucleic acid detection assays have become an essential and practical application of real-time PCR techniques, resulting in dramatic advances in molecular diagnostics^8,9^. However, no precise diagnostic assay has yet been approved by CE marked or the US FDA for detecting HIV-2 viral RNA.

**Figure 1:**
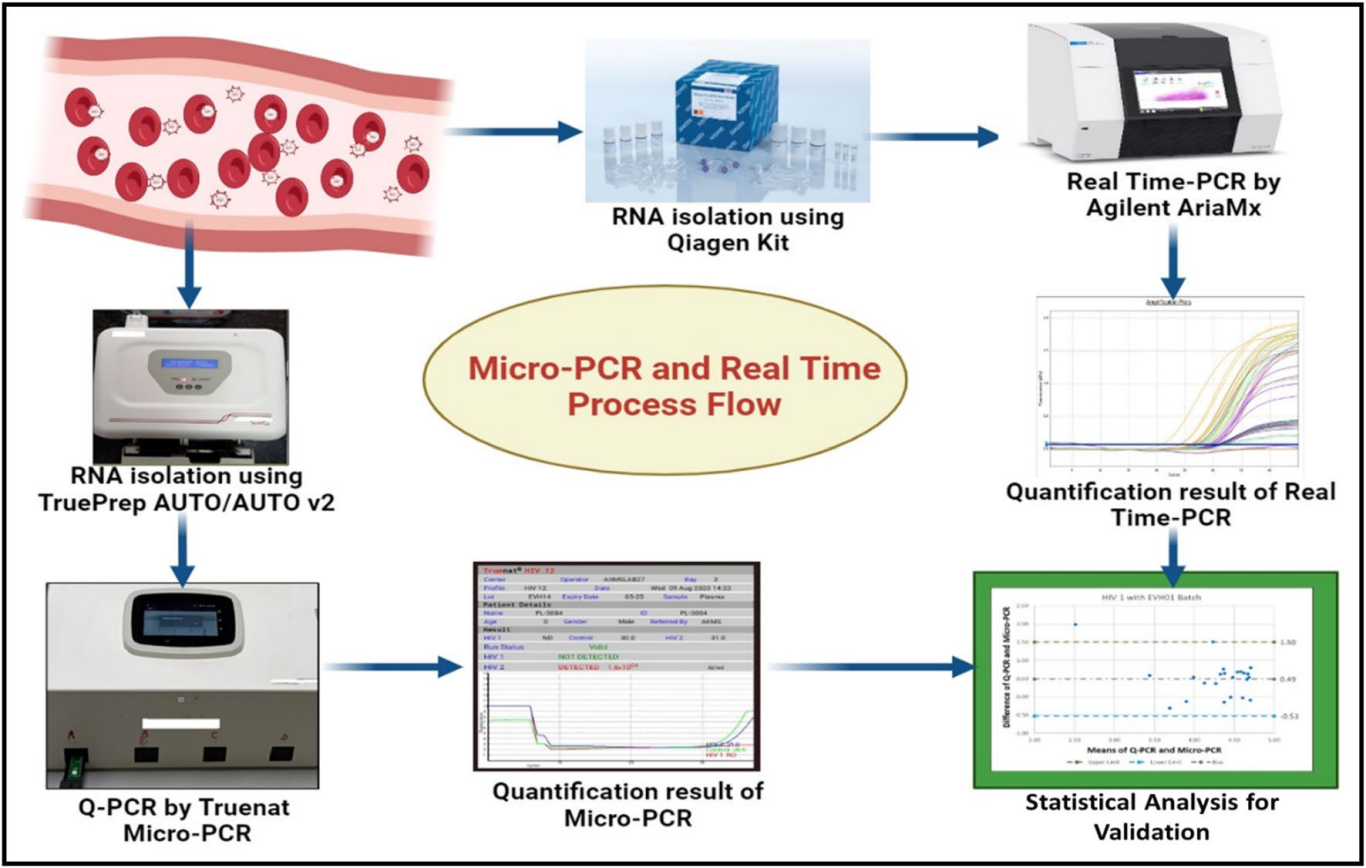
Flow chart of methodology for micro-PCR and real-time PCR. Blood samples were isolated for RNA using a Qiagen kit and TruePrep AUTO/AUTO v2 device. Both real-time PCR and Truenat™ micro-PCR utilise RNA for quantification. Statistical methods were used for the validation and interpretation of results.

However, most laboratories have developed the protocol for detecting HIV-2 RNA and are still sending the samples to national referral laboratories to confirm HIV reports as per the WHO consolidated guidelines^10–12^. Moreover, HIV RNA detection in plasma has enabled a new understanding and revolutionised the clinical setup of HIV-infected patients. Various HIV detection methods have been commercially developed and validated clinically^13,14^. Moreover, in-house methods for HIV-2 quantification by real-time PCR have been developed by the University of Washington and validated according to the College of American Pathology (CAP)^15^. Similarly, the public health agency of Canada has also developed HIV-2 viral quantification in digital droplet PCR (ddPCR) and validation done under the CAP regulations^15^. HIV-2 RNA kits are commercially available, such as Primer Design Limited, Southampton, UK, and Biocentric Bandel, France; they have been developed for research purposes only^16,17^. Unfortunately, due to the cost and technical constraints, patients living in countries with limited resources have not yet benefitted from molecular diagnostic tests^18^. They are restricted to centralised reference laboratories only. They have well-developed infrastructures and trained staff to perform real-time PCR for monitoring HIV-1/2 viral loads. Recently, Bigtec Labs, Bangalore, has developed a portable micro-PCR device, CHIP-based technology for detecting HIV-1 and HIV-2. The details of the processing steps are shown in Fig. 1. The significant key features of micro-PCR are rapid amplification, high sensitivity, and accurate quantification of HIV viral loads. The Micro-PCR system eliminates various issues related to HIV detection and monitoring in resource constrained situations by providing mobility, rapid results, less sample requirements, power efficiency, multiplexing capabilities, cost-effectiveness, and ease of use. This article describes a concise methodology for detecting HIV-1/2 viral load in patients utilising Micro-PCR technology and a statistical correlation with a previously established real-time PCR method. Therefore, for HIV prevention, treatment, and early care of HIV infection, urgently needs to assess the HIV-1 and HIV-2 quantification in blood samples.

## Methods

### Sample recruitment and collection

Three hundred subjects were screened from the Department of Medicine and HIV clinic AIIMS, New Delhi. Blood sampling was done from the Antiretroviral Therapy (ART) counters of the central collection facility, Department of Laboratory Medicine, AIIMS, New Delhi, for biochemical, serological and molecular tests. The AIIMS ethics committee approved the study, and informed consent was obtained from all the recruited patients. The methods employed in this study were conducted in accordance with the relevant guidelines and regulations as per the Declaration of Helsinki. EDTA and plain vials were used to collect 5.0 ml of blood samples, which were then centrifuged to separate the serum and plasma for processing. These samples were stored at − 80 °C till further processing steps. All subjects were screened for serology (anti-HCV antibody, IgM antiHBc, anti-HEV, HBsAg and HIV-1 &-2) and LFT (ALT, AST, Bilirubin). Serology of IgM antiHBc, anti-HEV and HBsAg was done using a commercially available ELISA kit (Bio-Rad Monalisa). HIV serology was done using the 4th generation ELISA kit (Genedia HIV Ag-Ab). In case the HIV serology is positive in patients, then patients were called for counselling and referred to the ART clinic, Department of Medicine, AIIMS, New Delhi, for further management of the patients. The case–control study involves 110 subjects divided into two groups: (I) HIV seropositive subjects (51 patients) and (II) Healthy subjects (59 patients). The LFT was done in the Department of Laboratory Medicine AIIMS, New Delhi. Out of 51 seropositive subjects, 26 for HIV-1 and 25 for HIV-2 were processed for further study.

### RNA isolation

**Figure 2:**
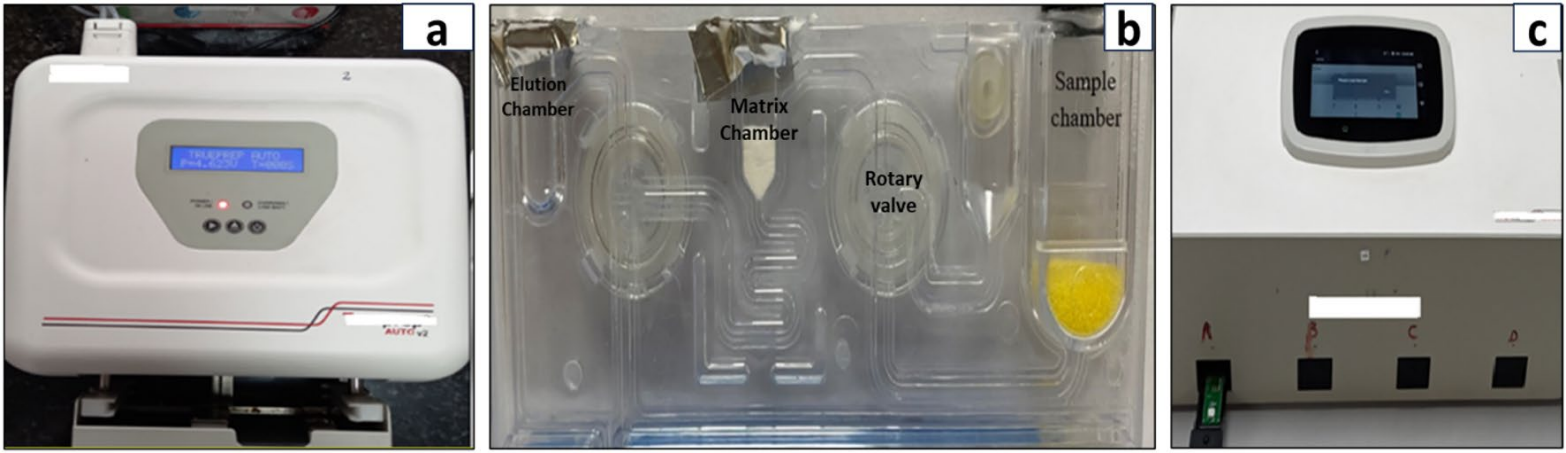
Extractor and Micro-PCR devices. (**a**) TruePrep auto extractor device used for the RNA isolation, (**b**) **cartridge** where the blood samples are processed and RNA isolated from blood and (**c**) **Chip-based RT-PCR** where 6 µl of RNA was loaded on Chip for quantification.

A total of 110 naive patients were included in the study by appropriate serological tests. The RNA from the patient sample is first extracted using TruePrep™ AUTO/AUTO v2 Universal cartridge-based sample prep device and TruePrep™ AUTO/AUTO v2 Universal cartridge-based sample prep kit according to the manufacturer’s protocol as shown in Fig. 2a, b. TruePrep™ uses the sample with a pretreatment reagent to automate the process of RNA isolation by lysing to the sample in the sample chamber, binding RNA to a solid support in a matrix chamber, and washing off the unwanted components followed by the elution of RNA from the solid support in the elution chamber. The yield of RNA concentration is obtained in the sample. The concentration of RNA was compared with the RNA obtained from the Qiagen isolation kit (Qiagen, Gmbh, Germany) by nanodrop spectrophotometer (Thermo Scientific, US).

### Real-timePCR

**Figure 3:**
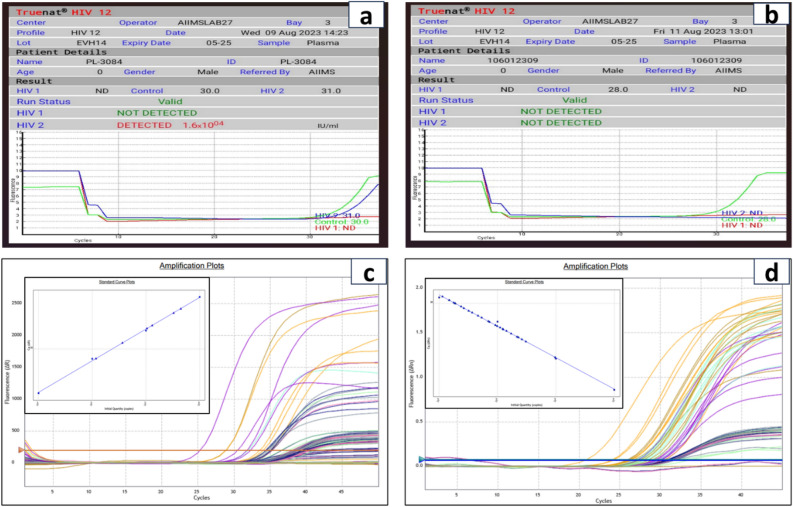
Quantitative PCR of HIV1-/2: (**a**) **Positive** and (**b**) **Negative samples**. The positive samples show “DETECTED” on-screen, while negative samples show “NOT DETECTED” status after quantitation. Amplification plots and standard curves generated by real-time PCR (Agilent AriaMx) for (**c**) **HIV-1** and (**d**) **HIV-2** positive samples are shown in the image. The amplification plot depicts the increase in fluorescence signal over PCR cycles, suggesting target DNA accumulation. The standard curve, which was created using known target concentration samples, facilitates in converting experimental Ct values to actual concentrations for precision measurement.

After the elution of RNA, the Truenat™ HIV-1/2 chip is placed on the real-time micro-PCR Analyzer chip tray, as shown in Fig. 2c. Six µL of the extracted RNA is dispensed into the microtube containing RT-PCR master mix reagents and kept for incubation at 60 s. Six µl of this solution is dispensed into the reaction well of the Truenat™ HIV-1/2 chip, wherein RNA is converted to cDNA by reverse transcription, followed by PCR amplification using specific target gene primers. Detection and monitoring were done in real-time using a TaqMan probe to analyse the amplification of the target genes in the real-time micro-PCR analyser (Bigtec Labs, Bangalore) against the internal controls. To provide a quantitative result, standard values (preset) for every batch are automatically compared to the Ct value of the test sample in the HIV-1/2 chip (Truenat™). A horizontal amplification curve is seen on the screen during the test run for negative samples without amplification (Fig. 3b). At the end of the test run, the HIV-1/2 “DETECTED” or “NOT DETECTED” result is shown in Fig. 3a. Moreover, in positive cases, the Ct values and International Units per millilitre (IU/ml) are displayed on the screen as shown in Fig. 3a.

The validity of the test run is also checked by the internal positive control (IPC), and the amplification is displayed on the device screen. The internal controls (positive and negative controls) were used from the Truenat® control panel II kit (MolBio Diagnostics, India). The eluted RNA was also amplified for HIV-1 (ARTUS, Qiagen, Gmbh Germany) and HIV-2 (ALTONA, Argentina) in real-time PCR (Agilent AriaMx) as per the manufacturer's protocol. The comparison of the viral levels (HIV-1/2) was done from the Truenat™ (micro PCR) device with the ARTUS and ALTONA kits (Agilent real-time PCR) in the same samples.

### Statistical analysis

The Bland–Altman plot represents the agreement between two paired variables measured on the same scale. It is applied to determine the agreement between two procedures or instruments in quantitative analysis. Also, it requires computing the difference between the two measurements (bias) and plotting it against their average. Therefore, the assumption of the two methods roughly represents the same results for each measured data. However, the limits of agreements have been used to determine the mean difference, which is approximately 1.96 times the standard deviation of the differences. Even a thorough assessment of the agreement requires a comparison of the two methodologies to make an understanding of any systematic or proportionate biases^19^. The significance of the tests was analysed using a student t-test. The scatter plot depicts the two quantitative relational variables for the same sample^20^. The scatter plot, combined with the Bland–Altman plot, visualises the two quantitative related variables for the same sample. This integrated methodology ensures a thorough review of method agreement, contributing to this study's outcomes and dependability.

### Ethics approval

The study was approved by the AIIMS Institutional Review Board of Ethics Committee (AIIMS, New Delhi, India). The ethical clearance number was IEC-270/01.04.2022, RP-08/2022, and dated 09.04.2022.

### Consent to participate

Written informed consent was obtained from each participant prior to recruiting in the study. All investigations and procedures were done as per standard guidelines.

## Results

**Figure 4:**
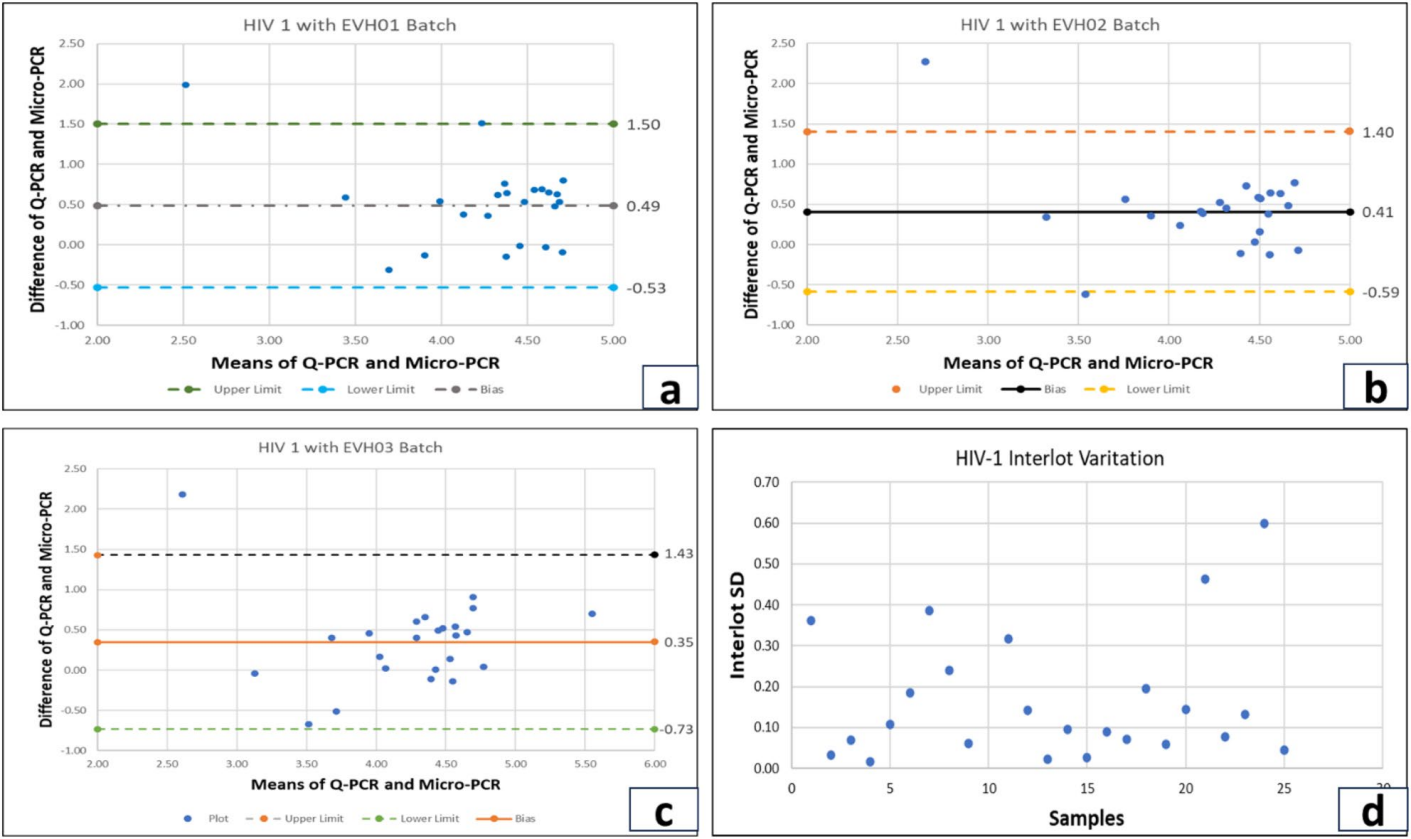
Degree of agreement in log copies/ml between viral loads determined by the Truenat PCR kit and Artus kit for HIV-1 virus. For Bland and Altman curves, the mean values for the sample obtained by the two techniques are plotted on the X-axis. The differences between the values obtained by the two techniques are plotted on the y-axis. The solid line shows the mean difference between the values, and the dotted lines show the mean difference plus or minus 1.96 SD (95% limits of agreement). The concordance of viral loads is assessed using the Truenat™ PCR and Artus kit, illustrated by Bland and Altman plots. For (**a**) **Device 1**, the upper limit is 1.5, and the lower limit is -0.53 (**b**) **Device 2**, the upper limit is 1.4, and the lower limit is -0.59. (**c**) **Device 3** shows an upper limit of 1.43 and a lower limit of -0.73. (**d) Interlot variation among devices**.

**Figure 5:**
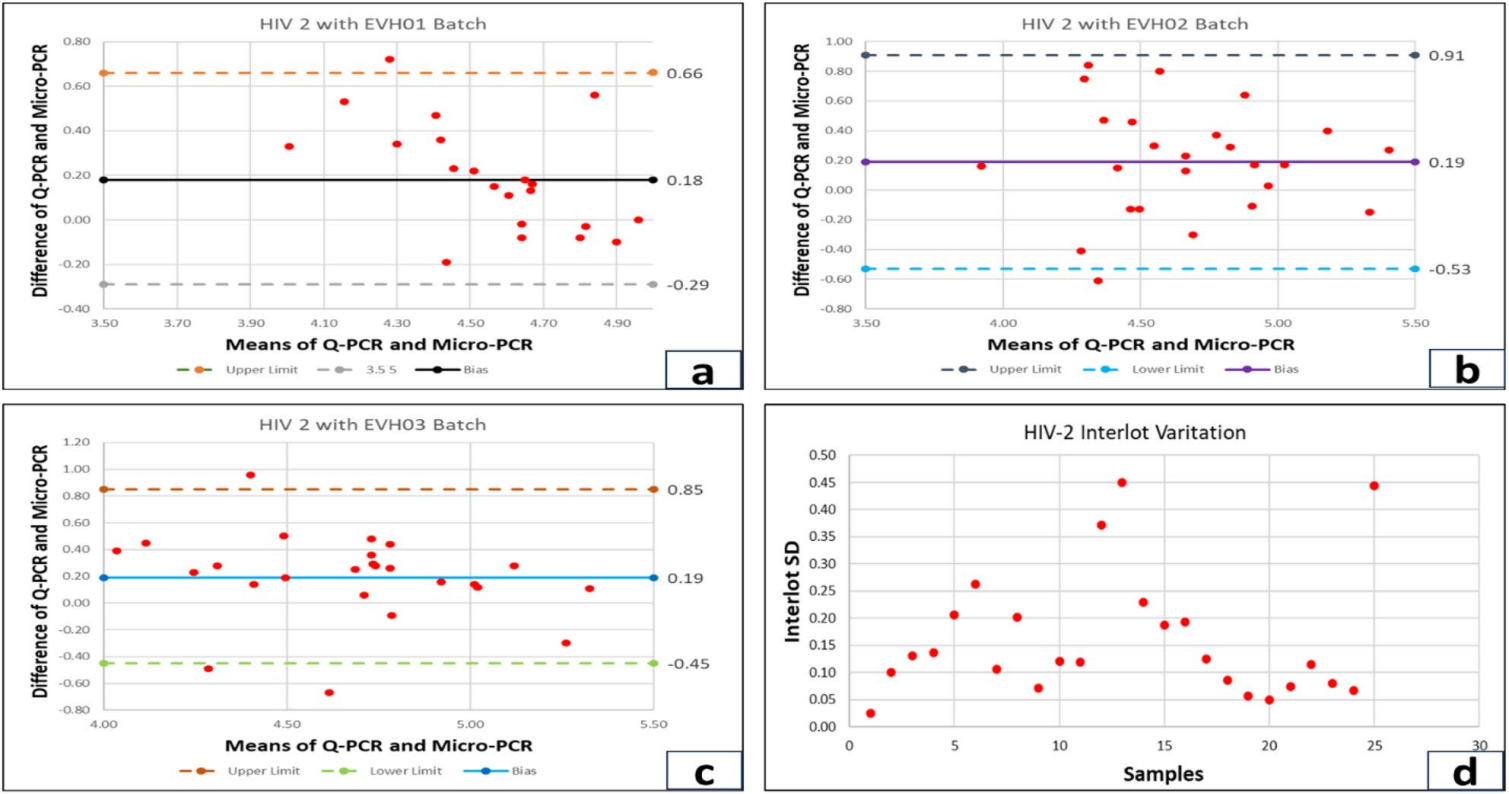
Degree of agreement in log copies/ml between viral loads determined by the Truenat™ PCR kit and Altona kit for HIV-2 virus. The concordance of viral loads is assessed using the Truenat^TM^ PCR and Artus kit, illustrated by Bland and Altman plots. For (**a) Device 1**, the upper limit is 0.66, and the lower limit is -0.29 (**b) Device 2**, the upper limit is 0.91, and the lower limit is -0.53. (**c**)** Device 3** shows an upper limit of 0.85 and a lower limit of -0.45. (**d) Interlot variation among devices**.

**Figure 6:**
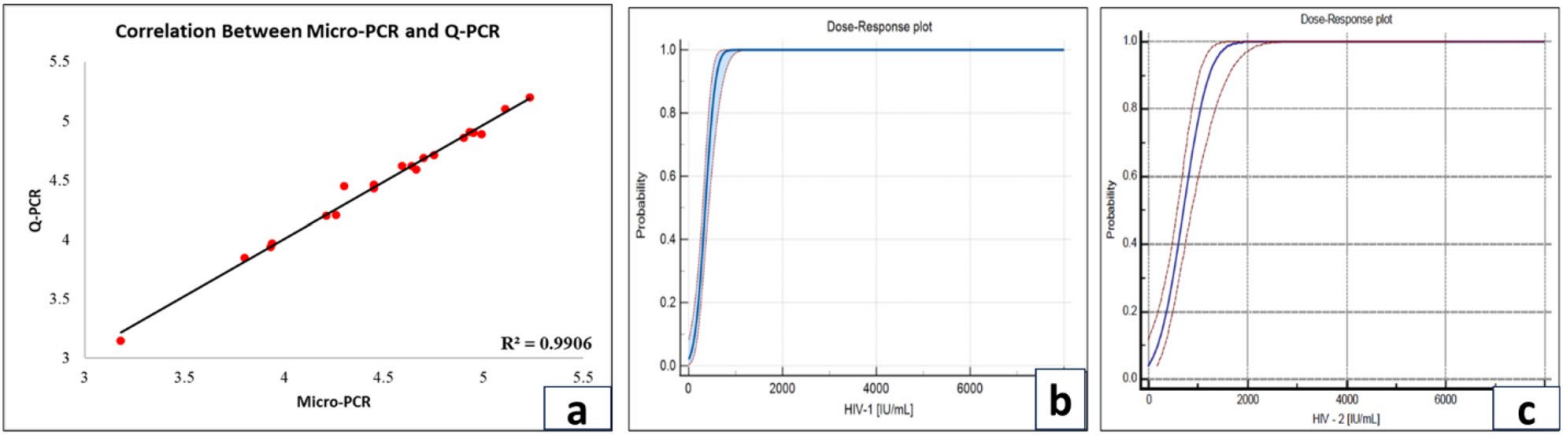
(**a**) A scatter plot comparing the results of micro-PCR and Q-PCR is shown in the figure, suggesting a strong correlation between the two procedures, as indicated by the correlation coefficient (R^2^ value) of 0.99. This indicates a high degree of agreement or similarity in the findings achieved by both approaches. A dose-response chart for (**b**) **HIV-1** and (**c**) **HIV-2**, which shows how the assay's sensitivity varies with varying doses. The primary goal is to determine each virus's Limit of Detection (LOD), providing important information regarding the assay's capacity to detect low amounts of HIV-1 and HIV-2.

To study the viral RNA load in HIV infected patients, we tested the samples from 110 subjects. After the confirmation of HIV serology, viral RNA was isolated using a Qiagen Kit and then underwent real-time PCR for the target gene amplification. NIBSC (WHO) standards were used for HIV-1/2 while processing the reaction for real-time PCR. Compared to the Qiagen kit for viral extraction and real-time PCR, Truenat™ has a turnaround time of less than 1.5 h and 1500 copies/ml of plasma LOD. One hundred ten samples were processed to assess the sensitivity and specificity of the Truenat™ for HIV-1/2 assay. The reaction assay was performed in Agilent AriaMx real-time PCR system for Qiagen extracted RNA samples. The amplification plot of HIV-1 and HIV-2 positive samples is shown in Fig. 3c, d. Extracted RNA was quantified using the Truenat™ micro-PCR system. The accuracy of the Truenat™ HIV-1/2 assay was determined by comparing the ARTUS HIV-1 RG RT-PCR kit for HIV-1 and the ALTONA real star HIV-2 RT-PCR kit for HIV-2. Bland-Altmann analysis was done to compare methods and correlation between micro-PCR and real-time PCR.The results are indicated in Fig. 4a, b, c. The viral load was compared with three micro-PCR devices (EVH01, EVH02, EVH03). The mean difference between Artus and different lots of Truenat™ tests is 0.4 log IU/ml for EVH01, 0.41 log IU/ml for EVH02 and 0.36 log IU/ml for EVH03, respectively. These values were less than the clinically significant difference of 0.5 log IU/ml. All samples were run in triplicates with the three different devices. The inter-lot variation of viral load determinationis shown in Fig. 4d. Moreover, the average deviation was similar with other lots of Truenat™ (0.13 log IU/ml), which was in the acceptable range as these values are less than 0.5 log IU/ml. However, the mean difference between the Altona kit and the different lots of Truenat™ HIV-2 levels were 0.16 log IU/ml for EVH01, 0.17 log IU/ml for EVH02 and 0.19 log IU/ml for EVH03, respectively. They were also found to have less than 0.5 log IU/ml difference, as shown in Fig. 5a, b, c. The Interlot variation in HIV-2 viral load estimation is shown in Fig. 5d. The average deviation in viral load estimation between different lots of Truenat™ HIV-1/2 tests was 0.13 log IU/ml and 0.24 log IU/ml, respectively. It was also acceptable as these values are less than a clinically significant variation of 0.5log IU/ml. The correlation coefficient (r) for the micro-PCR and Q-PCR is 99%, which had a very strong relationship between these two methods, as shown in Fig. 6a. The sensitivity, specificity and accuracy were ~ 100% (95% CI 86.77–100%), ~ 100% (95% CI 93.94–100%) and ~ 100% (95% CI 95.77–100%), respectively between Truenat™ viral load and Artus HIV-1 Q-PCR viral load in the same samples as shown in Table 1. Similarly, the sensitivity, specificity and accuracy were ~ 100% (95% CI 86.28–100%), ~ 100% (95% CI 93.94–100%) and ~ 100% (95%CI 95.77–100%), respectively, between Truenat™ viral load and Altona HIV-2 Q-PCR viral load in the same samples as shown in Table 2.

**Table 1:** Sensitivity, specificity and accuracy of HIV-1 by micro-PCR.

Detection of HIV-1 in Truenat HIV-1/-2 & Artus HIVirus 1 RG Q-PCR tests				
	Artus HIVirus 1 RG and Q-PCR for HIV-1			
		Positive	Negative	Total
Truenat HIV-1/2	Positive	26	0	26
	Negative	0	59	59
	Total	26	59	85

**Table 2:** Sensitivity, specificity and accuracy of HIV-2 by micro-PCR.

Detection of HIV-2 in Truenat HIV-1/2 & Altona real star HIV-2 RT-PCR tests				
	Altona real star HIV-2 RTRT- PCR kit 1.0			
		Positive	Negative	Total
Truenat HIV-1/2	Positive	25	0	25
	Negative	0	59	59
	Total	25	59	84

### Accuracy of truenat™ HIV-1 and HIV-2 assay

Accuracy was determined by RNA extraction and Truenat™ HIV-1/2 PCR for different sample titers for five consecutive days. The accuracy of the Truenat™ HIV-1/2 assay, which quantitatively estimates the amount of HIV-1 and HIV-2 virus using three different lots of reagents, was also determined compared to Artus HIV-1 RG Q-PCR and Altona real star HIV-2 RT-PCR assay kit. The Bland–Altman plot of the method comparison shows that the mean difference between the tests is 0.23 Log IU/mL, less than the clinically significant difference of 0.5 Log IU/mL. The Correlation coefficient (r) for the micro-PCR Truenat™ method is 99%, which shows a strong relationship between the methods. The micro-PCR Truenat™ reaction was performed on three devices of a different lot to find the inter-lot deviation, and the average deviation in HIV-1 RNA and HIV-2 RNA estimated was 0.37 IU/mL and 0.45 IU/ml, respectively. This is acceptable as the values are less than the clinically significant variation of 0.5 IU/mL in both cases.

### Linearity

The linearity analysis was performed according to the CLSI guidelines. Serial HIV-1 and HIV-2 RNA dilutions were performed from 1.0 × 10^8^ copies/ml to 1.0 × 10^2^ copies/ml. Nucleic acids were extracted three times with TruePrep™ Auto Sample Prep for each dilution, followed by PCR on Truenat™ (TruePrep™ AUTO/AUTO v2). For HIV-1 and HIV-2 RNA, the assay is linear over eight digits [1.0 × 10^8^ copies / mL to 1.0 × 10^2^ copies/mL]and [1.0 × 10^8^ copies/mL to 1.0 × 10^2^ copies/mL], respectively.

### Limit of detection

The LOD was determined by testing dilutions of HIV 1 and HIV-2 NIBSC International Standard diluted in Negative human plasma. The evaluation was performed according to CLSI guidelines. Probit analysis of the data was used to determine the concentration of the RNA that could be detected with a positivity rate of 95%, as shown in Fig. 6b, c. The LOD for HIV-1 with NIBSC 4th International Standard was found to be 635 IU/mL (95% CI 533–826), and the LOD for HIV-2 with 2nd WHO International Standard was found to be 1372.80 IU/mL (95% CI 1134.17–1836.01) by the Truenat™ HIV-1/HIV-2 test.

### Precision

Accuracy was tested using the Truenat™ HIV Assay for High, Medium and Low titers of HIV-1 and HIV-2 RNA were administered for five consecutive days. Each day, PCR was performed twice for each HIV-1/2 RNA titer. The standard deviations were within the 0.5 log IU/mL range the Truenat™ HIV-1/2 assay allowed.

### Robustness

Potential sample carryover within the Truenat™ HIV-1 and HIV-2 assay was assessed by alternating positive and negative samples. The number of samples run was 10 positive and 10 negative. The results showed that there was no carryover contamination.

### Clinical specificity

The 59 negative runs are correlated between the methods and show 100% specificity for the Truenat™ HIV-1 and HIV-2 assay.

### Analytical specificity (cross-reactivity)

The cross-reactivity of the test was evaluated using samples negative to HBsAg, anti-HCV, coinfection of HCV & HIV both and HBV-RNA markers but positive to the virus that may have cross-reactivity or coinfected with HIV-1/2. Ten serum samples positive for HCV RNA and 5 serums for co-infection of HCV and HIV RNA were analysed in the cross-reactivity study.

### Clinical sensitivity

Positive samples (HIV-1: 25 and HIV-2: 26) were run and correlated between the methods, resulting in 100% sensitivity to the Truenat™ HIV-1/2 assay.

### Concordance

A satisfactory agreement [> 95% within log variation] was observed between the viral load determined by the Truenat™ HIV-1/2 assay and the Artus and Altona internal HIV assay.

## Discussion

In this study, we have compared the simultaneous detection of HIV-1 and HIV-2 in the Truenat™ system, as previously, there were no platforms available for detecting both Viral loads in a single reaction. However, the measuring of HIV-1 and HIV-2 viral load is essential for monitoring HIV infected patients. There is an increasing tendency to understand the pathophysiology of nucleoside or nucleotide analogue drugs, but it still needs to be clarified to define the viral load dynamics^21^. HIV viral load monitoring can predict the suppression of the immune system; therefore, the patients have compromised immune effector functions and antibody-dependent cytotoxicity (ADCC) activity^22^.

Therefore, developing new-generation, dual-target nucleic acid testing (NAT) assays is an essential step for improving the accuracy of HIV assays and limiting the risk of under-quantification^8^. These new assays must accurately detect and quantify HIV-1/2 RNA in samples, exhibit a lower limit of detection (LLOD) and lower limit of quantification (LLOQ) values comparable with the currently available tests, and show good analytical performance^23^. A susceptible, specific, and robust micro-PCR device for quantification, targeting the detection of both HIV-1 and HIV-2, has been developed that can simultaneously determine viral load in HIV infected individuals. In addition, rapid release of results is required for treating and monitoring patients under ART^14,24^. We have isolated the viral RNA from TruePrep™ AUTO/AUTO v2 Universal cartridge-based sample prep extractor device and Qiagen RNA kit. The TruePrep™ Auto is an electromechanical device with a 2-line LCD screen showing RNA isolation status. This device is designed for gradual heat, mixing, and adding reagents to the cartridge's contents as it is placed into the holder.  Subsequently, nucleic acid materials are eluted in the Elution chamber. The RNA quality and quantity were almost equal in both the isolation processes after the measurement from the Nanodrop spectrophotometer. Truenat™ is a novel portable real-time micro-PCR similar to real-time PCR (Agilent). This device works on chip-based containing dried MgCl_2_, internal controls and dried RT-PCR reagents for real-time amplification to detect the HIV-1/2 virus. The Truenat™ HIV-1/2 chip also stores information about the used chip to prevent any accidental reuse of the chip.110 patients were evaluated from both (micro-PCR and real-time PCR) devices, and our results had higher precision, specificity and linearity across a wide dynamic range for HIV-1 and HIV-2 viral loads. Bland-Altmann plot analysis showed that the viral loads from Truenat^TM^ PCR meet with the real-time PCR. The Bland-Altmann mean difference for the comparison was 0.36–0.41 log IU/ml from the micro-PCR devices for the HIV-1 virus, and for the HIV-2 virus, the mean differences were from 0.16 to 0.19 log IU/ml. In addition, the Pearson correlation coefficient value shows a strong correlation between these two devices. Our results have shown 100% sensitivity and specificity compared to real-time PCR. Notably, the tests showed good linearity and reproducibility in triplicates from 2 to 6 log copies/mL^25^, relevant ranges for clinical decision making. This accurate test is thus reliable for treatment monitoring of patients with HIV.

Researchers have attempted many methods to simplify PCR and real-time PCR platforms to develop nucleic acid amplification^26^. Recently, micro-PCR Truenat^TM^ was used to detect HBV viral loads, diagnose HBV infection, and monitor drug efficacy^27^. Micro-PCR Truenat™ was also reported for the detection of M. tuberculosis, Malaria, Papillomavirus, Beta-CoV and SARS-CoV-2 with asensitivity of 94.7% (95% CI 89.8, 97.6), 99.3% (95% CI 95.5, 99.9), 97.5% (95% CI 86.8, 99.9) and 100% respectively^28–31^.

Furthermore, the focus must be heavily oriented to reach this technology to remote areas, which will help to assess early detection of care for multimorbidity and good quality of life. Studies have shown that HIV-positive patients are more prone to develop cardiovascular diseases and preserved ejection fraction^32–36^. Overall, this device will help to monitor the viral load of HIV-1/2 and provide insight into developing therapeutic and preventive vaccines, novel immunotherapies and an early cure for HIV infected patients.

## Limitation and future

This device has limitations, particularly where the patient number is higher, as this device can run a maximum of 4 samples in a batch. In this study, we found that the HIV detection limit for HIV-1 and HIV-2 was 635 and 1372.8 IU/mL. It requires further research and validation up to a lower detection limit. Multicentric studies are required to address the challenges of variable mutations in the population.

## Conclusion

The healthcare system needs to address the challenges of HIV infection where the detection facility is not available, particularly in remote areas. Our study suggests that this device is cost-effective, easy to handle, battery-operated, accurate, and can be performed for routine uses in remote places and places where well-developed infrastructure and sophisticated equipment are unavailable. Overall, TruePerp™ and Truenat™ devices can detect both HIV-1 and HIV-2 in a single reaction. To the best of our knowledge, this is the novel portable system for quantitating nucleic acids.

## Data Availability

The corresponding author, Shyam Prakash, will provide data supporting the research findings upon a feasible request.
